# Effects of High-Frequency Transcranial Magnetic Stimulation for Cognitive Deficit in Schizophrenia: A Meta-Analysis

**DOI:** 10.3389/fpsyt.2019.00135

**Published:** 2019-03-29

**Authors:** Yi Jiang, Zhiwei Guo, Guoqiang Xing, Lin He, Haitao Peng, Fei Du, Morgan A. McClure, Qiwen Mu

**Affiliations:** ^1^Department of Radiology and Imaging Institute of Rehabilitation and Development of Brain Function, The Second Clinical Medical College of North Sichuan Medical College Nanchong Central Hospital, Nanchong, China; ^2^Department of Psychiatry, Harvard Medical School, Belmont, CA, United States; ^3^Department of Radiology, Peking University Third Hospital, Beijing, China

**Keywords:** transcranial magnetic stimulation, high-frequency, schizophrenia, cognition, working memory

## Abstract

**Objective:** Repetitive transcranial magnetic stimulation (rTMS) has been applied to dorsolateral prefrontal cortex (DLPFC) to improve cognitive function of patients with schizophrenia (SZs). The aim of this meta-analysis was to evaluate whether a high-frequency rTMS course could enhance cognitive function in SZs.

**Methods:** Studies published in PubMed, Cochrane Library, Embase, ScienceDirect, and Web of science were searched until April 2018. The search terms included: “repetitive transcranial magnetic stimulation” or “Rtms,” “SZ,” or “schizophrenia,” and “neuro-cognition” or “neurocognitive performance” or “cognitive effects” or “cognitive” or “cognition” or “working memory” or “executive function” or “language function” or “processing speed,” After screening the literatures according to inclusion and exclusion criteria, extracting data, and evaluating the methodological quality of the included studies, a meta-analysis was performed using RevMan 5.3 software (The Cochrane Collaboration, USA).

**Results:** A total of 9 studies on cognitive dysfunction of SZs were included and involved 351 patients. A significant efficacy of high-frequency rTMS on working memory in SZs was found compared to sham stimulation [*p* = 0.009, standardized mean difference (SMD) = 0.34]. Specifically, rTMS treatment positioned on the left DLPFC, with a total pluses <30,000 was more significantly more effective in improving the working memory (SMD = 0.33, *p* = 0.03). No improvement was found in other cognitive domains such as executive function, attention, processing speed, and language function. For the follow-up observations, high-frequency rTMS had long-lasting sustained effects on working memory (SMD = 0.45, *p* = 0.01) and language function (SMD = 0.77, *p* = 0.02) in SZs.

**Conclusions:** High-frequency rTMS over the left DLPFC with a total pulses <30,000 stimulation could significantly improve working memory in SZs for an extended period of time.

## Introduction

As a major psychiatric disorder, schizophrenia has a prevalence of 1.0 % of the global population (1). The clinical symptoms of schizophrenia can be classified into three major categories: negative symptoms, positive symptoms, and cognitive deficits (2, 3). Cognitive deficits are a manifestation of brain dysfunction and the most prominent symptom of patients with schizophrenia (SZs), which usually precedes other symptoms and is associated with poor drug adherence and recurrence of first episode psychosis (4, 5). Multiple cognitive deficits in SZs include cognitive processes such as attention, working memory, and executive function are correlated with the dysfunctions of distinct brain regions (6). For example, several previous studies (7, 8) have shown that frontal lobe, temporal lobe, parietal lobe, and hippocampus were the core areas associated with cognitive deficits in SZs. Further functional magnetic resonance imaging (fMRI) study showed a decrease in the volume of the prefrontal cortex of SZs compared with that of healthy controls (HCs) (9). In addition, animal model studies have shown the involvement of synaptic plasticity, demyelination, and white matter lesions in the development of cognitive deficits in SZs (10). However, so far, the pathological mechanism of cognitive deficits in SZs is still unclear. Additionally, classic antipsychotic drugs could alleviate the positive and negative symptoms of SZs, but not cognitive deficits (11). Therefore, there is a need to investigate new and effective therapeutic methods for cognitive dysfunction of SZs.

Repetitive transcranial magnetic stimulation (rTMS) is a safe and non-invasive therapy that induces currents in local areas of the cerebral cortex through rapidly changing magnetic fields to alter neuronal activity in the cerebral cortex. It can alter the excitability of the cerebral cortex, brain's metabolic activity, neural electrophysiological activity, neuronal plasticity, local brain function, and functional connectivity in different brain regions (12), and these may be the main mechanisms of rTMS-ameliorated cognitive function in psychiatric patients. The TMS stimuli can be delivered in different patterns which is often divided into “regular” and “patterned” protocols. Regular rTMS refers to stimulation at constant frequencies, and is always divided into high frequency rTMS (HF-rTMS, stimulation at > 1.0 Hz) and low frequency rTMS (LF-rTMS, stimulation at ≤ 1.0 Hz). Patterned rTMS refers to the application of repeated busts of rTMS pulses with a certain frequency separated by various interburst intervals, such as theta burst stimulation (TBS) including continuous TBS (cTBS) and intermittent TBS (iTBS), which are different from the regular rTMS. So far, rTMS has been widely applied to the treatment of depression, generalized anxiety, post-traumatic stress disorder, Parkinson's disease and chronic pain, and has become a new prospective therapy for psychiatric disorders.

rTMS has been applied to negative symptoms (13) and auditory hallucinations (14, 15) in SZs. Currently, the focus of rTMS research on SZs have been switched from auditory hallucinations and negative symptoms to cognitive deficits. In general, high-frequency rTMS contributes to cortical excitability (16, 17), while low-frequency rTMS inhibits cortical excitability (18). The efficacy of high-frequency rTMS in promoting cognitive functions of various cognitive disorders has been demonstrated in several studies (19, 20). Moreover, one meta-analysis reported that high-frequency applied to left DLPFC was more effective in improving cognitive function of psychiatric disorders (21). However, it is still too early to draw a definitive conclusion regarding the efficacy of high-frequency rTMS on the cognitive deficits of SZs. One reason is that there is still no systematic and detailed meta-analysis to verify the efficacy of rTMS. Another important reason is the current controversy over the clinical effect of rTMS. For example, while some studies have observed a significant steady effect of rTMS therapy on SZs (22, 23), others found no improvement in cognitive deficits after rTMS treatment in SZs (13, 24). Therefore, in this meta-analysis, we evaluated studies of high-frequency rTMS on cognitive function in SZs to determine if rTMS treatment was an effective therapy and which parameters were more appropriate.

## Methods

### Database Search

We performed the meta-analysis of the data from the PubMed, Cochrane Library, Embase, Sciencedirect, and Web of science published before April 2018. The search terms of “repetitive transcranial magnetic stimulation” or “rTMS”, “SZ”, or “schizophrenia” and “neuro-cognition” or “neurocognitive performance” or “cognitive effects” or “cognitive” or “cognition” or “working memory” or “executive function” or “language function” or “processing speed” were used. The following inclusion criteria were used for the meta-analysis: (a) treatment with rTMS; (b) use of rTMS more than one session; (c) subjects were schizophrenic; (d) use of high frequency rTMS (>1.0 Hz); (e) more than five patients per study; (f) sham stimulation was used as a comparator; (g) papers published in English.

### Quality Assessment

The quality of each study was assessed for the risk of bias in randomized trials by using the Cochrane collaboration tool. The risk of Cochrane's collaborative bias assessment tool evaluated seven specific types of research bias, including random sequence generation, allocation concealment, blinding of participants, and researchers, results evaluation blindness, incomplete result data, selection outcome reports, and other sources of bias.

### Data Extraction

The following data were acquired from the studies: (a) the sample size of real rTMS group and sham rTMS group; (b) the mean and standard deviation (SD) of the cognitive measurements after treatment and follow-up; (c) the changes of reaction time and correct rate in after rTMS treatment of different cognitive scales; (d) study design and treatment parameter; (e) clinical and treatment characteristics (education, course of disease, localization of treatment, frequency, intensity, number of treatment session).

### Categorization of Cognitive Measurements

Due to the wide range of scales involved in the cognitive domain, using a single scale to detect rTMS treatment for cognition may not be meaningful. Moreover, there was no unified standard for the classification of cognitive domain. Duchek (25) proposed that cognitive dysfunction may include the impairment of the following cognitive aspects: language understanding and generation, pattern recognition, task organization, reasoning, attention, and memory. According to the main aspects involved in the intervention, Cicerone et al. (26) classified cognitive rehabilitation into seven categories: attention, visual perception, and constructive ability, verbal communication, memory, question resolution, and executive function, multiple model interventions, and comprehensive cognitive rehabilitation. Except for these seven cognitive categories, another recent study of this author (27) believed that integrative holistic cognitive rehabilitation separately should also be the cognitive domain.

Based on these classifications and cognitive domains mentioned in the included studies, five categories involving executive function, working memory, processing speed, attention, as well as language function were analyzed in this meta-analysis as shown in Table 1. Execution function includes the ability to form goals, to plan how to achieve goals, and to perform mental tasks effectively, including scales such as Wisconsin Card Sorting Test, Stroop Test, Trail Making Test B. Working memory is the core memory impairment, which is a system for temporary storage and processing of limited information and has functions for maintaining, refreshing, and converting information, including the California Verbal Learning Test, Digit Span Backwards at al. Processing speed generally refers to the speed with which the brain processes information, as well as cognitive flexibility. The scales are as follows: Trail Making Test A, Digit Symbol Substitution Test, Number Connection Task. Attention is the orientation and concentration of mental activity or consciousness on certain objects, including scales such as D2 attention task. Language function refers to aphasia and language fluency including scales such as Phonemic Verbal Fluency, Semantic Verbal Fluency. Due to the diversity in the scale, we used the similar scale as much as possible in the same cognitive domain.

**Table 1:** Cognitive scales included in the analyses. K, Number of Studies.

**Cognitive domain**	**Cognitive scales**	**K**
Executive function	Wisconsin card sorting test (categories)	2
	Wisconsin card sorting test (correct rate total error)	2
	Wisconsin card sorting test (perseverative answers)	2
	Tower of London	2
	Trail making test B	4
	Controlled oral word association test	2
	Stroop test	1
	Grooved pegboard	1
Processing Speed	Trail making test A	4
	Digit symbol substitution test	1
	Number connection task	1
	Symbol coding	1
	Token motor total	1
Working Memory	KAI short test of general intelligence	1
	Rey auditory verbal learning test	1
	Verbal learning test, recall	2
	Verbal learning test, delayed recall	2
	Face recognition	1
	Digit span backwards	1
	N-back accuracy (3 back)	1
	Digit sequencing	1
	Verbal memory	1
Attention	D2 attention task	2
	N-back accuracy (1 back)	1
	Digit span forward	1
Language Function	Phonemic verbal fluency	1
	Semantic verbal fluency	1
	Regensburg word fluency test	1
	Controlled oral word association test	2
	Semantic and letter fluency	1
	Mehrfachwahl-Wortschatztes	2

### Statistical Analysis

Meta-analysis was performed using RevMan 5.3 software (The Cochrane Collaboration, USA). The normalized standardized mean difference (SMD) was used as the effect indicator with 95% confidence interval (CI). The effect model was determined by the heterogeneity which was assessed by using the Cochran's Q statistic and *I*^2^ test. When heterogeneity was not significant [Q-statistics (*p* > 0.05) or when *I*^2^ < 50%], a fixed effect model was applied. If not (*I*^2^≥50%), a random effect model was used. The funnel diagram was conducted to examine the bias of publication. In addition, some subgroup analysis was conducted based on the clinical parameters.

## Result

### Information of Included Studies

**Figure 1:**
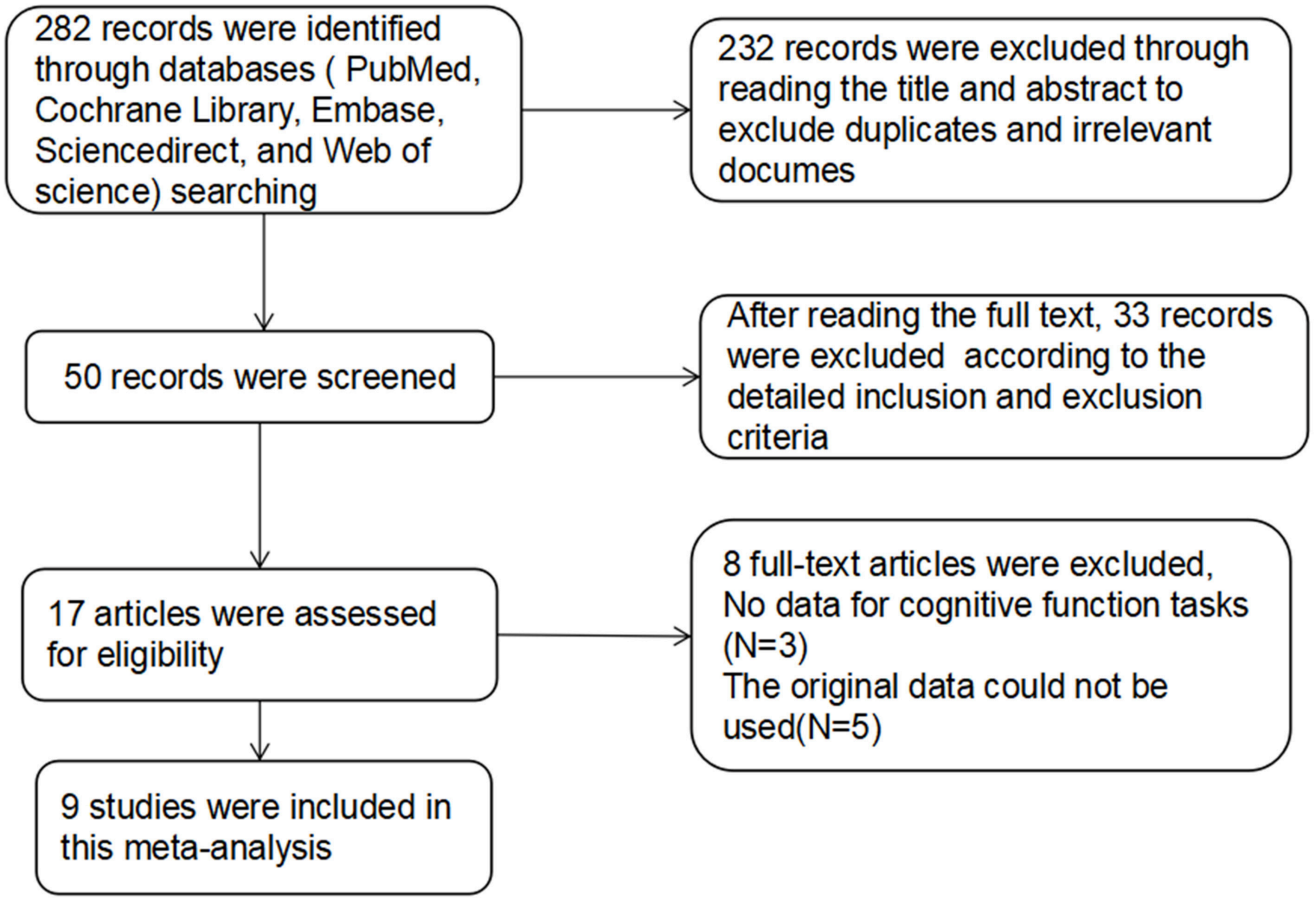
Document screening process and results.

For studies with high-frequency rTMS treatment in SZs, the database search yielded 282 publications. After reviewing the titles and abstracts, excluding duplicates and irrelevant documents, 50 articles remained potentially eligible. After reading the full text, only 17 articles met the inclusion criteria, in which the original data of 5 studies could not be used, 3 studies did not provide the raw data. The remaining 9 articles (28–36) were used for data extraction (Figure 1). The detailed treatment parameters and clinical characteristics in the article were shown in Table 2.

**Table 2:** Characteristics of included studies.

**Study**	**Real/Sham**	**Disease duration (year)**	**Total pulses**	**Intensity (%MT)**	**DLPFC site**	**Frequency (Hz)**	**No.of sessions**	**Follow-up (day)**
Hason et al. (28)	77/79	–	15,000	110	Left	10	15	105
Mogg et al. (29)	8/9	25/9	20,000	110	Left	10	10	14
Dlabac-de Lange et al. (30)	16/16	15.67/9.92	60,000	90	Bilateral	10	15	28
Dlabac-de Lange et al. (31)	11/13	16/7.5	60,000	90	Bilateral	10	15	
Mittrach et al. (32)	18/14	5.7/5.6	10,000	110	Left	10	10	
Barr et al. (33)	13/14	18.62/24.5	30,000	90	Bilateral	20	20	
Rollnink et al. (34)	6/6	–	8,000	80	Left	20	10	
Wölwer et al. (35)	18/14	5.7/5.6	10,000	110	Left	10	10	
Francis et al. (36)	9/10	23.4/22.3	12,000	110	Bilateral	20	10	14

### Quality of The Studies

**Figure 2:**
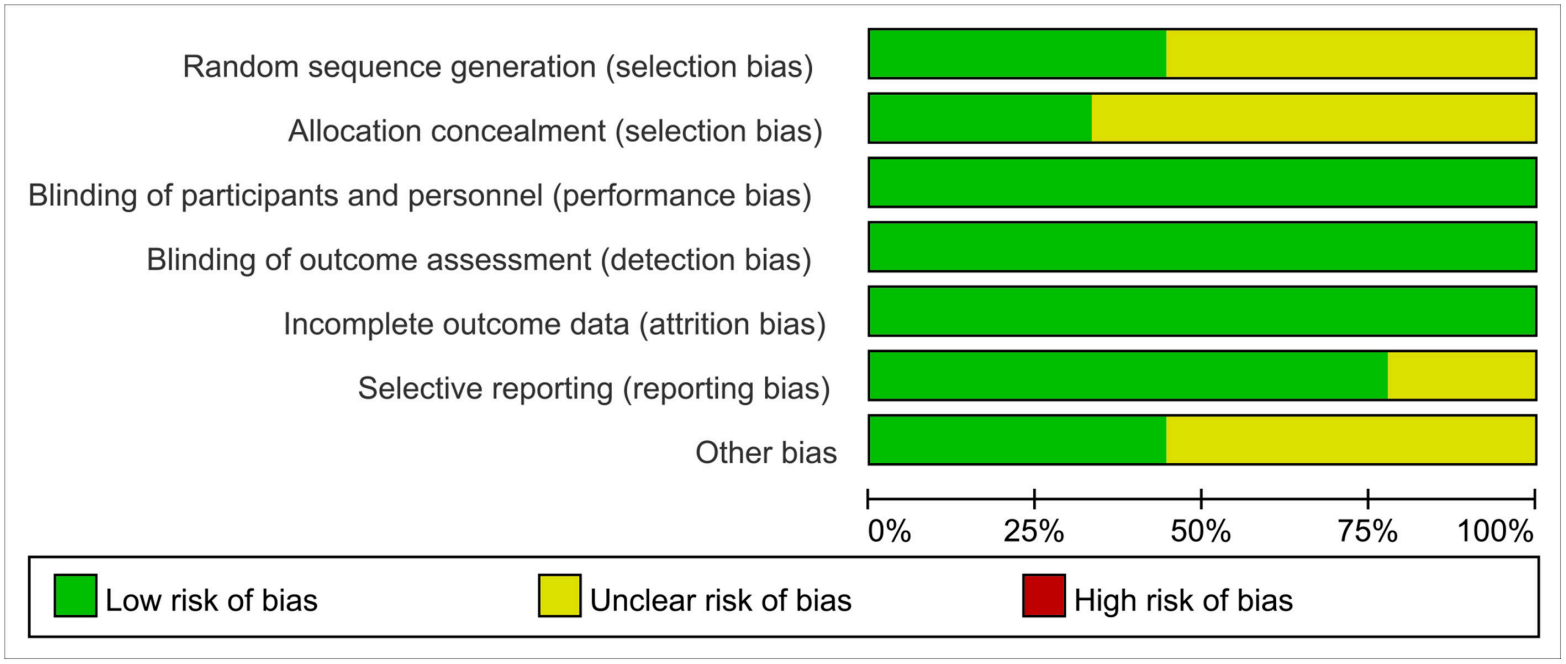
The assessment of bias risk in the 9 studies included in the quantitative synthesis.

Reviewers evaluated the risk of bias for the nine studies by using Cochrane Collaboration guidelines. In blinding of participants, blinding of outcome assessment, publication bias, and incomplete outcome data, the vast majority of standards were low-risk. However, most studies had an unclear risk of bias for allocation concealment (Figure 2).

### Acute Effects of rTMS Treatment on Various Cognitive Domains

**Figure 3:**
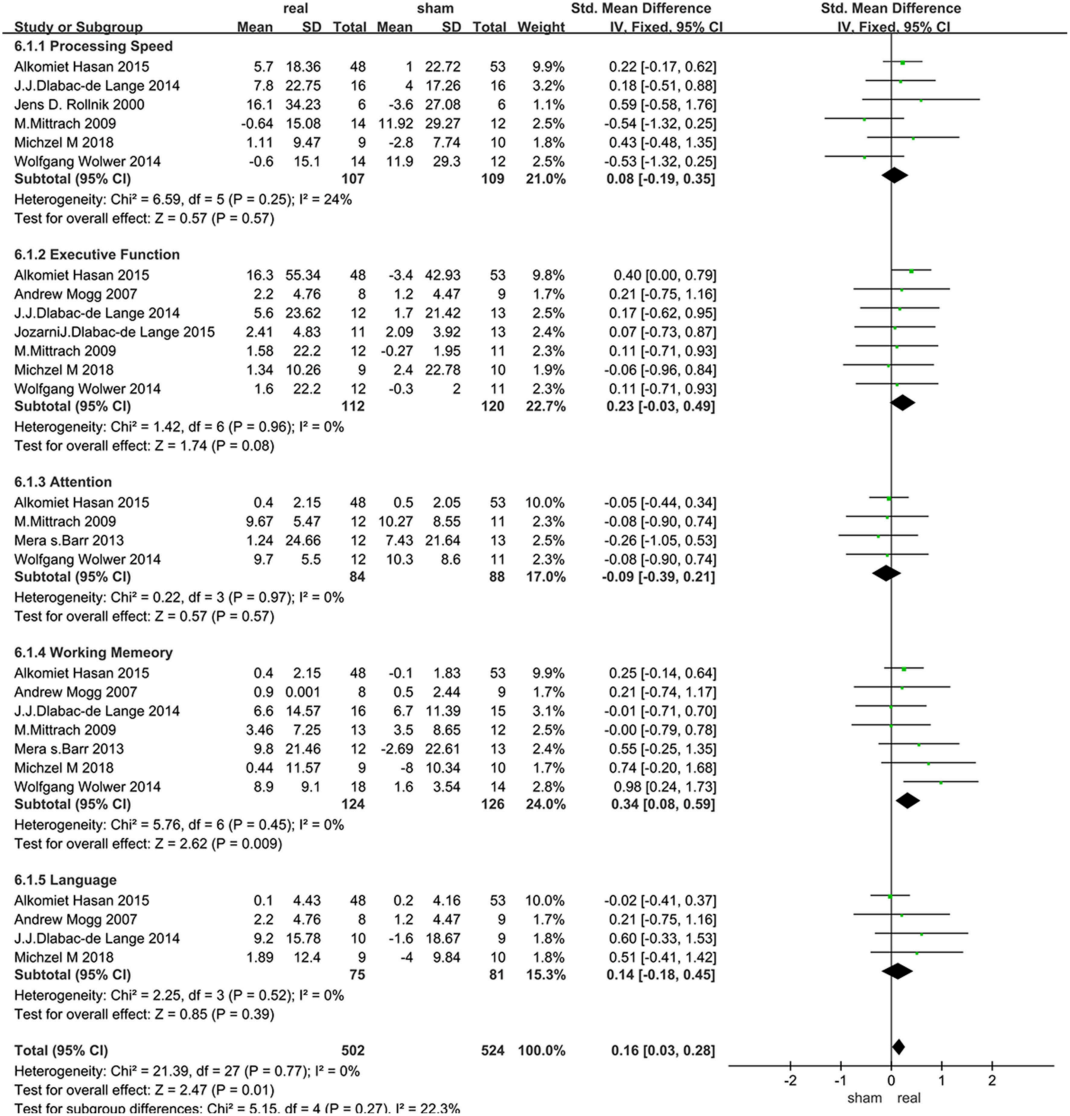
Immediate efficacy in various cognitive domains after rTMS treatment.

**Figure 4:**
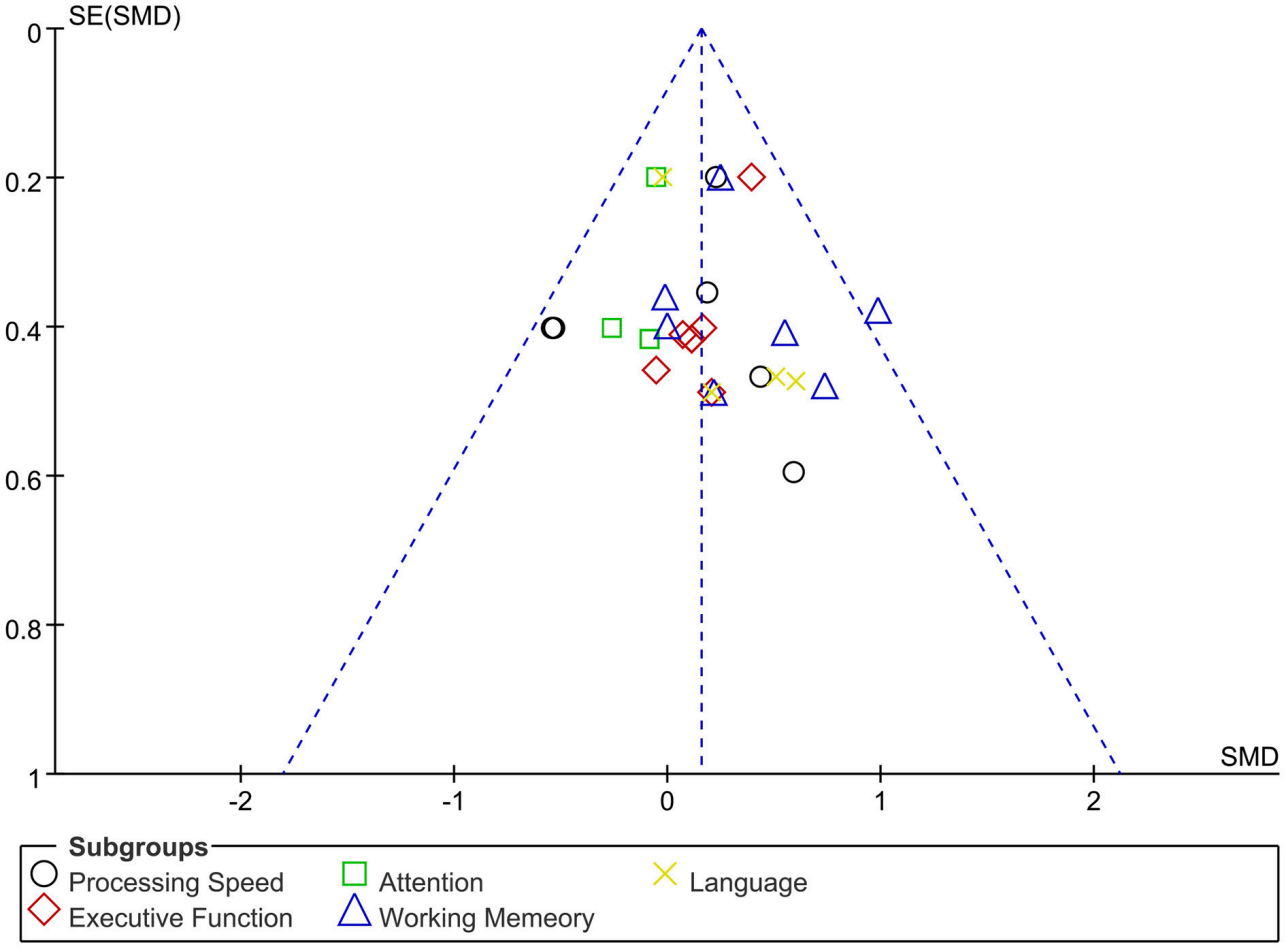
Dissemination bias of immediate effects in various cognitive domains after rTMS treatment.

The assessment score changes post-treatment relative to baseline data were extracted for acute effects. Due to heterogeneity *I*^2^ < 50%, the fixed effect mode was selected. The results of various cognitive domains were shown in Figure 3. Furthermore, to assess the existence of publication bias in these studies, the funnel diagram of the meta-analysis was shown in Figure 4. The scattered points of the included research effects were evenly distributed, and formed an inverted funnel shape symmetrically around the center line, indicating that there was no obvious publication bias in all studies.

### Processing Speed

Data related to rTMS treatment for processing speed were analyzed for 6 studies that included 216 patients. The results showed no significant difference between rTMS stimulation and sham stimulation post rTMS (Figure 3).

### Executive Function

Data of executive function were analyzed for 7 studies that included a total of 232 patients. The result showed that rTMS stimulation improved executive function at a trend of level compared with the sham stimulation (SMD = 0.23, 95% CI, −0.03–0.49, *P* = 0.08) (Figure 3).

### Attention

Data of rTMS treatment on attention were analyzed for 4 studies that included a total of 172 patients. The result showed no significant difference between rTMS stimulation and sham stimulation (Figure 3).

### Working Memory

**Figure 5:**
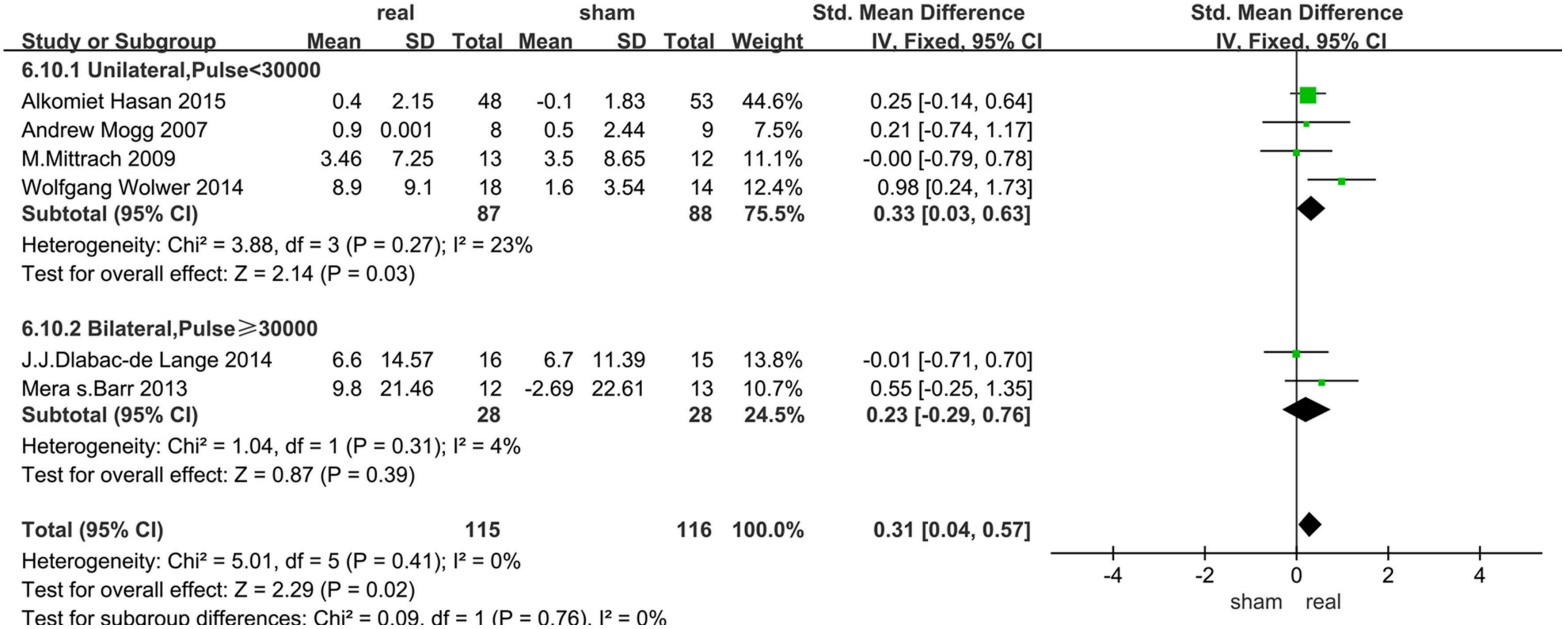
Immediate efficacy of working memory subgroup analysis after rTMS treatment.

Data of rTMS treatment on working memory were analyzed for 7 studies, which included a total of 250 patients. The result showed that the effect of rTMS stimulation was significantly better than sham stimulation in improving working memory (SMD = 0.34, 95% CI, 0.08–0.59, *P* = 0.009), and without heterogeneity found between the trials (*I*^2^ = 0%) (Figure 3). Subgroup analysis, based on the treatment parameters of stimulation site and total number of pulses, showed a significant effects of rTMS on working memory (SMD = 0.33, 95% CI, 0.03–0.63, *P* = 0.03), without significant heterogeneity (*I*^2^ = 23%), when rTMS was applied to left DLPFC and the total pulses were < 30,000 (Figure 5).

### Language Function

Data of rTMS treatment on language function were analyzed for 4 studies which included a total of 156 patients. The result showed no significant difference between rTMS stimulation and sham stimulation (Figure 3).

### Long-Term Efficacy of rTMS Treatment in Various Cognitive Domains

**Figure 6:**
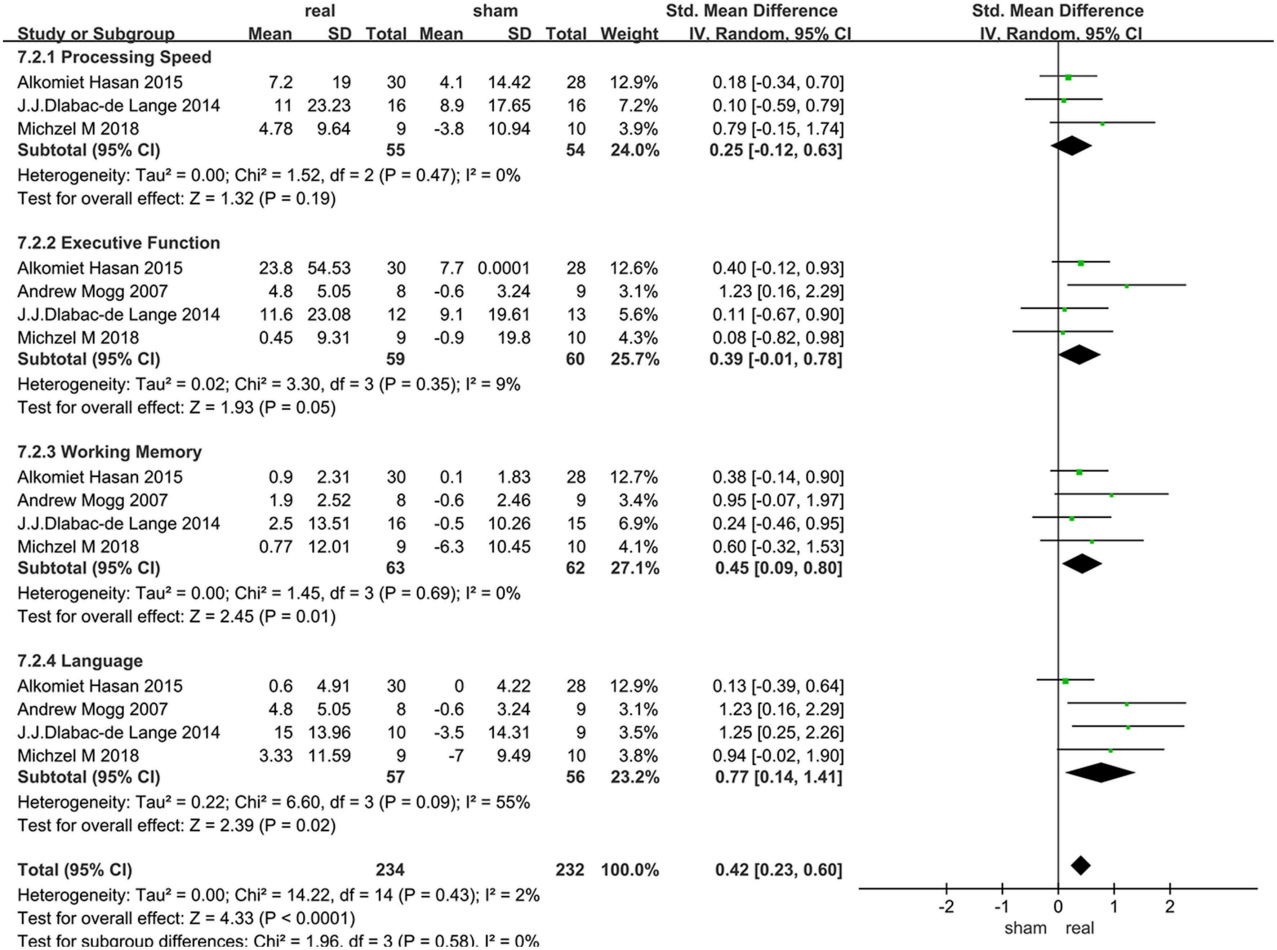
Long-term follow-up efficacy in various cognitive domains after rTMS treatment.

Only 4 articles with follow-up data were included, 2 of which only followed up patients for 2 weeks, and the others followed up patients for 1 months and 3 months, respectively. Due to the heterogeneity of language function within the group (*I*^2^ = 55%), random effect model were used. Various cognitive domains results were shown in Figure 6.

### Processing Speed

Data of rTMS treatment on processing speed from 3 studies were analyzed, which included a total of 109 patients. There was no significant difference between rTMS and the sham operations during follow-up (Figure 6).

### Executive Function

Data of rTMS treatment on executive function from 4 studies were analyzed, which included a total of 119 patients. There was no significant difference between rTMS and the sham operations during follow-up (Figure 6).

### Working Memory

Data of rTMS treatment on working memory from 4 studies were analyzed, which included a total of 125 patients. The result showed that rTMS stimulation had a significant long-term efficacy compared to sham-stimulation (SMD = 0.45, 95% CI, 0.09–0.80, *P* = 0.01) (Figure 6).

### Language Function

Data of rTMS treatment on language function from 4 studies were analyzed, which included a total of 113 patients. rTMS stimulation has produced significant long-term efficacy compared to sham stimulation (SMD = 0.77, 95% CI, 0.14–1.41, *P* = 0.02) (Figure 6).

## Discussion

Although there were several earlier meta-analyses published on the effects of rTMS on the cognitive domain in psychiatric disorders, there was no specific and detailed systematic review on SZs. To our best knowledge, this study was the first meta-analysis on rTMS-treated cognitive domain in SZs. Our results supported the value and efficacy of high-frequency rTMS on improving the cognitive deficits in SZs, especially in working memory (both acute and long-term effects).

### Acute Effects of rTMS Treatment on Various Cognitive Domains

This meta-analysis of rTMS revealed a significant main effect size of rTMS on working memory in SZs, which was consistent with the results of Guse et al. (23) In fact, for the cognitive improvement, the similar report happened to other mental illness meta-analysis, for example Liao et al. (37) showed that high-frequency rTMS could represent a promising therapeutic method for improving the impaired cognitive function in dementia patients. Even in normal people, Moser et al. (38) performed rTMS stimulation (20 Hz and 80% MT) on DLPFC in 19 patients with dysfunction showed that the rTMS had significant effects. The exact mechanism of rTMS in the treatment of cognitive impairment is still not clear. But as far as we know rTMS could induce currents in local areas of the cerebral cortex through rapidly changing magnetic fields to reshape local brain function and functional connectivity in different brain regions. In recent years, with the development of neuroanatomical and functional imaging studies, the mystery of rTMS in the treatment of mental diseases has been further uncovered. Multiple studies (39, 40) have shown that dopamine (DA) neurotransmitters in SZs were disordered. The activity of DA neurons is regulated by activation and inhibition pathways in the prefrontal cortex, which is related to cognitive deficit. In fact, studies (41, 42) have found that activation in the prefrontal cortex can regulate the release of dopamine by rTMS, which may be the basis for treating cognitive deficit of SZs. In addition, most of the patients enrolled in this study had negative symptoms, the improvement of negative symptoms of SZs treated with high-frequency rTMS may improve the cognitive ability.

rTMS showed positive effects on executive function and working memory (Figure 3), but only the improvement of working memory reached a statistically significant level. As the higher level of cognitive functions, both the executive function and working memory are related to DLPFC. But still some differences exist between them, which could be explained by the following reasons. Firstly, the executive function includes higher cognitive processes such as problem solving, mental planning, initiation, and suppression behavior. The main function of the execution system is to monitor the process of cognition and the flexible dynamic adjustment resulting from environmental changes. This adaptive behavior requires a flexible brain activity that is responsible for maintaining and updating current relevant information, and controls the perception of input information and the execution of output behavior through top-down control (23, 43, 44). However, working memory is usually operated through continuous performance tasks that require permanent maintenance and operation of input information. Executive function may involve more interconnected brain areas and require more specific activity on adjustment rTMS effectively modifies. In addition, from the perspective of anatomy, neuroimaging study has identified DLPFC, which was involved in the preservation and extraction of activated information and was the key region responsible for functional memory dysfunction in SZs (45), as damaging to the frontal lobe lead to learning and memory impairment in SZs (46). All of these may account for the more significant improvements in working memory, but the connotation of different mechanisms in the cognitive domains and the intersection of their anatomy need to be further researched.

DLPFC was also indispensable for other multiple cognitive domains, including ability of language generation, transfer and response suppression, as well as working memory, most of which were included in this meta-analysis. In this study, we did not observe any significant effects of rTMS on other cognitive processes such as attention, processing speeds, and language function compared to sham stimuli in SZs. This suggested that rTMS did not have an up-regulation effects on universal DLPFC function. Similar results were found in two previous systematic review/meta-analysis of psychiatric disorders (21, 47). It is known that functional relevant brain regions for attention and processing speed were mainly related to the parietal and temporal lobes. Previous studies (48, 49) have suggested that the subparietal lobes in the cerebral cortex dominate in attentional orientation and damaging to the parietal lobe caused neglect of the visual space (50). Even Hilgetag et al. (51) found that TMS can affect the early processing of visual spatial attention, and ipsilateral parietal lobe activity was enhanced by rTMS stimulation of the contralateral parietal lobe. For language function, the main areas of the language brain were in the lower part of the frontal gyrus (52, 53). Neuroimaging studies (54, 55) showed that specific dysfunctions in the dorsal and ventral regions were associated with the poor language function. Furthermore, recent study (56) has shown that selective impairment in the prefrontal and temporal cortex and language dominant hemispheres may all cause language disorders in SZs. Targeting only DLPFC may not improve the cognitive impairments related to other regions.

The subgroup analysis of working memory showed that the unilateral stimulation site (left DLPFC) with a total number of pulses of < 30,000 was significantly effective in improving cognitive disorders in SZs. In fact, previous fMRI studies have demonstrated functional specificity of distinct brain regions, which highlighted the regional specificity and the importance of accurate rTMS localization. The DLPFC as an important target, has differentially distributed functional connectivity networks, and precise positioning of rTMS on DLPFC had a different impact on efficacy (57, 58). For our results, stimulating the left DLPFC was more effective than bilateral stimulation, a result similar to the reports on other cognitive diseases (59–61). Firstly the activities of the left prefrontal lobe, thalamus in the sub-cortical loop of schizophrenic patients were reduced after rTMS stimulation when compared with normal subjects (9). Furthermore, an fMRI study (62) of a delayed word-memory task (CPWd), showed that the activation of left PFC was reduced in patients with SZs during the identification phase of this task, which suggested that high-frequency rTMS to left DLPFC was a key target for the treatment of working memory in SZs. Secondly, abnormal development of the lateral hemisphere may be the cause of SZs, that is, the development of the right and left hemispheres of SZs are disturbed in the embryonic stage. The white matter of the frontal lobe of a healthy person has an asymmetrical state, that is, the left side is greater than the right side (63). In contrast, such brain lateralization is absent in SZs (64). By enhancing the activity of the left DLPFC to achieve lateralization may be a mechanism of improved working memory after rTMS.

The total number of pulses of rTMS is another important parameter, which is the number of treatment days multiplied by the daily pulses. The daily pulses and methodology used in most high-frequency rTMS studies were similar, yet varied in the total number of stimulation. It is not clear how the stimulation protocols are optimized or selected. Based on the data of the meta-analyses of other cognitive diseases, rTMS was more effective when the total treatment time was < 4 weeks and the daily treatment pulses < 1,200 (65), that was, the total number of pulses was < 28,800, similar to our findings in SZs (30,000). In addition, other meta-analysis (66) showed that a greater number of stimuli will not produce better outcome.

### Long-Term Effects of rTMS Treatment for in Various Cognitive Domains

So far the long-term effect of rTMS on cognitive function in SZs is still unknown. Systematic investigation of rTMS-induced long-term cognitive improvement is lacking. Our results indicated that high-frequency rTMS can induce a long-term significant improvement in these cognitive processes: working memory and language function. It is thought that rTMS can promote nerve remodeling, promote the regeneration of damaged peripheral nerves and restore conduction function better than local electrical stimulation, promote brain cell proliferation, and improve nerve function. With these assumptions, one study (67) demonstrated rTMS-induced long-term potentiation (LTP) of neural responses in humans, which may be the reason why cognitive improvement was sustainable over the long term. But a link between LTP and duration of cognitive improvement is still unknown. Most rTMS studies are short-term or limited to several months of follow-up observation. A 3-year long-term follow-up study showed beneficial effects on adolescent depression and cognitive impairment after rTMS (68). Another reason for the significant improvement in the cognitive function of follow-up after rTMS treatment may be due to the confounding effects of learning acquired during the follow-up (28). The results of improvements in language function at follow-up were surprising, even though the language areas of the brain were not located at DLPFC. On the one hand, it is probable that stimulation of the DLPFC not only impact on the cortical excitability within the stimulated cortex but also leads to excitability changes of the whole circuitry. On the other hand, only four articles contained follow-up data, and the time was short and varied. Therefore, in the future, it is hoped that more research involved follow-up investigations are needed to support our views.

But some limitations exist in this study. Firstly, the inclusion of a wide range of cognitive domain and a large number of scales may lead to variations in the classification of cognitive functions. Secondly, the relatively few of rTMS studies on cognition of SZs was a limiting factor. Last, the pathological mechanism and symptoms of SZs were heterogeneous and complicated that may affect the results.

## Conclusion

High-frequency rTMS could improve cognitive performance in SZs, especially on working memory, when targeting left DLPFC with a total number of pulses < 30,000. In addition, high-frequency rTMS has a long-lasting effects on working memory and language function.

## Author Contributions

All authors listed have made a substantial, direct and intellectual contribution to the work, and approved it for publication. YJ and ZG contributed to the design, analysis, and drafting of the work. GX, LH, and HP performed the data acquisition and interpretation of the work. FD and MAM contributed to the revision for important intellectual content of the article and the final approval of the version to be published. QM reviewed and contributed to the full manuscript.

### Conflict of Interest Statement

GX was employed by company Lotus Biotech.com LLC. The remaining authors declare that the research was conducted in the absence of any commercial or financial relationships that could be construed as a potential conflict of interest.
